# Robust knowledge-guided biclustering for multi-omics data

**DOI:** 10.1093/bib/bbad446

**Published:** 2023-12-06

**Authors:** Qiyiwen Zhang, Changgee Chang, Qi Long

**Affiliations:** Department of Biostatistics, Epidemiology and Informatics, University of Pennsylvania, 19104, PA, USA; Department of Biostatistics and Health Data Science, Indiana University School of Medicine, Indianapolis, IN 46202, USA; Department of Biostatistics, Epidemiology and Informatics, University of Pennsylvania, 19104, PA, USA

**Keywords:** biclustering, bayesian hierarchical model, multi-view data, MCMC

## Abstract

Biclustering is a useful method for simultaneously grouping samples and features and has been applied across various biomedical data types. However, most existing biclustering methods lack the ability to integratively analyze multi-modal data such as multi-omics data such as genome, transcriptome and epigenome. Moreover, the potential of leveraging biological knowledge represented by graphs, which has been demonstrated to be beneficial in various statistical tasks such as variable selection and prediction, remains largely untapped in the context of biclustering. To address both, we propose a novel Bayesian biclustering method called Bayesian graph-guided biclustering (BGB). Specifically, we introduce a new hierarchical sparsity-inducing prior to effectively incorporate biological graph information and establish a unified framework to model multi-view data. We develop an efficient Markov chain Monte Carlo algorithm to conduct posterior sampling and inference. Extensive simulations and real data analysis show that BGB outperforms other popular biclustering methods. Notably, BGB is robust in terms of utilizing biological knowledge and has the capability to reveal biologically meaningful information from heterogeneous multi-modal data.

## INTRODUCTION

Biclustering is a popular statistical approach for simultaneously grouping both rows (features) and columns (samples) of a data matrix. By organizing the data into sub-matrices that contain similar elements, biclustering has gained significant traction, particularly in high-dimensional settings where only a limited number of features contribute significantly to differentiating various clusters. A prominent application of biclustering lies in complex disease subtyping, which aims to identify coherent subgroups of individuals based on specific biological features. Numerous studies have demonstrated the capacity of biclustering to unveil crucial biological insights, thus offering interpretable solutions [1, 2].

The existing biclustering methods can be categorized into four classes: (i) probabilistic and generative methods including FABIA [3], SSBi [4] and SSLB [5]; (ii) matrix factorization approaches including SSVD [6] and iSSVD [7]; (iii) combinatorial methods including OPSM [8] and ISA [9] and (iv) neural networks including BARTMAP [10] and AD [11]. The SSLB used Spike-and-Slab Lasso priors to induce adaptive shrinkage on both factor and loading matrices. However, it is specifically designed for single-view data and lacks the ability to harness crucial information from diverse yet related data, such as multi-omics data. The iSSVD, based on singular value decomposition, is a frequentist method that incorporates stability selection [12] to control Type I error rates when detecting biclusters. While it allows for the analysis of multi-view data, it does not accommodate discrete data types, such as read counts in RNA-Seq, single nucleotide polymorphisms (SNPs) and copy number data. The ISA employs the greedy algorithm to identify submatrices with all rows and columns above a pre-specified threshold, which can significantly effect the biclustering results and their interpretation. We refer readers to [13] for more detailed reviews of biclustering methods.

However, none of the aforementioned biclustering methods have utilized biological graph knowledge, despite evidence demonstrating the effective improvement in variable selection performance across different statistical tasks [14, 15]. An example of such biological knowledge is gene regulatory networks, which provide valuable information regarding regulators and their potential targets. Numerous public resources, such as Kyoto Encyclopedia of Genes and Genomes (KEGG; [16]), Gene Ontology (GO; [17]) and Mummichog [18], collect and store biological graph information. A recently proposed probabilistic biclustering method, known as GBC [19], developed hierarchical shrinkage priors to incorporate biological graph information. Although GBC demonstrates promising performance compared with other methods lacking graph incorporation, we found a limitation in their graph-incorporated priors. Specifically, their priors require the inverse covariance matrix of the shrinkage parameters, which imposes the graph information, to be diagonal dominant. This restriction crucially hampers the efficient use of graph information, especially when the underlying network grows larger.

In this article, we propose a novel Bayesian graph-guided biclustering (BGB) method, which comprises three key components. First, the BGB prior formulation effectively addresses the diagonal-dominant issue of GBC, enabling more efficient utilization of graph information. The proposed priors offer explicit control over the correlation level of the shrinkage parameters and adaptability to different prior knowledge. This flexibility empowers the model to handle scenarios involving larger network sizes or constructed networks containing noise. Second, the BGB, using the data augmentation technique in [20], facilitates a unified framework capable of handling multi-modal inputs that may consist of diverse data types, such as Gaussian, binomial and negative binomial data. Third, the proposed method allows for the retrieval of overlapped biclusters, while a majority of existing biclustering methods [21, 22] assume orthogonality among resulting biclusters. This is a crucial capability in applications where biclustering is frequently applied, such as cancer sub-typing studies. In these contexts, the same gene might be co-active in multiple subtypes.

The rest of the paper is organized as follows. Section 2 introduces the proposed method. Section 3 presents an efficient Markov chain Monte Carlo (MCMC). In Section 4, we conduct simulation studies to assess the performance of our method in comparison with the existing methods. In Section 5, the performance of the proposed method is evaluated on the real data. We conclude with a brief discussion in Section 6.

## METHODOLOGY

### Model framework

Suppose $X=[(X^{(1)})^T,\dots ,(X^{(H)})^T]^T$ is the data matrix consisting of $H$ modalities, where $X^{(h)}$ is related to the $h$th modality with $p_h$ features and $n$ samples. The total number of features is $p=\sum _{h=1}^{H} p_h$. The proposed method aims to identify submatrices of $X$ that correspond to the biclusters containing similar elements $x_{ji}$. Formally, a bicluster is defined by subsets of rows (features) and columns (samples) for which the columns exhibit the similar behavior on the corresponding subset of rows and vice versa. Such a submatrix manifests as an outer product $\boldsymbol{w}\boldsymbol{z}^T$ of two vectors $\boldsymbol{w}$ and $\boldsymbol{z}$. Building on this insight, we consider a multiplicative bicluster model with the following structure: 


(1) \begin{align*}& \mu = \boldsymbol{m}\boldsymbol{1}^T+\sum_{l=1}^{L} \boldsymbol{w}^l(\boldsymbol{z}^l)^T =\boldsymbol{m}\boldsymbol{1}^T + WZ,\end{align*}

where $\mu $ relates to the expectation of $X$ through the link functions depending on the data types and $\boldsymbol{1}$ is the vector with each entry equal to $1$. $\boldsymbol{m} \in \mathbb{R}^{p\times 1}$, $W=[\boldsymbol{w}^1,\dots ,\boldsymbol{w}^L]\in \mathbb{R}^{p\times L}$ and $Z^T=[\boldsymbol{z}^1,\dots ,\boldsymbol{z}^L] \in \mathbb{R}^{n\times L}$ refer to the location vector, factor loading matrix and latent factor matrix, respectively. We follow the convention that the superscript (subscript) refers to the column (row). The number of biclustering $L$ is allowed to be unknown and is learnt through the model selection procedure. We assume that both the factors and loading are sparse.

One important property of the proposed model is its incorporation of biological graph information which is denoted as $\mathcal{G} = \langle P,E \rangle $, with a set of nodes (features) $P$ and a set of edges $E$, representing the interactions between these features. We assume that there is no edge across modalities. A matrix is considered compatible with the graph $\mathcal{G}$ if its off-diagonal entries exhibit the same zero/non-zero pattern as the adjacency matrix of $\mathcal{G}$. Intuitively, the incorporation of the biological graph information helps reduce false negatives and false positives in variable selection. For example, if the $j$th feature and $k$th feature are (not necessarily directly) connected, there is a higher chance that these two features will be selected or not selected simultaneously for a given latent factor. This is reflected in the factor loading matrix $W$, where $w_{jl}$ and $w_{kl}$ tend to be either zero or non-zero for a specific $1 \leq l \leq L$.

The likelihood function is assumed to be conditionally independent as follows: 


\begin{align*} & \pi(X|\mu) = \Pi_{j}\Pi_i \pi_j(x_{ji}|\mu_{ji}) \text{ with}\ 1 \leq j\leq p, 1 \leq i \leq n, \end{align*}

where $\pi _j(\cdot )$ refers to the density function for the $j$th variable. A number of distributions can be considered as the model inputs including the Gaussian, binomial and negative binomial distribution. For Gaussian data, the likelihood is $ \frac{(\rho _{j})^{1/2}}{\sqrt{2\pi }}e^{-\rho _j(x_{ji}- \mu _{ji})^2/2}$ with the precision $\rho _{ji} \equiv \rho _{j}$. For binomial data, the likelihood is ${n_j \choose x_{ji}} \frac{e^{\mu _{ji}x_{ji}}}{(1+e^{\mu _{ji}})^{n_j}}$ with the number of trials $n_{ji} \equiv n_j $ and $x_{ji}\in \{0,1,\dots ,n_j\}$. For negative binomial data, the likelihood is ${r_j+x_{ji}-1 \choose x_{ji}}\frac{e^{\mu _{ji}x_{ji}}}{(1+e^{\mu _{ji}})^{r_j+x_{ji}}}$ with the failure parameter with $r_{ji} \equiv r_j$ and the non-negative integer $x_{ji}$. The probability $p_{ji}$ of binomial density or negative binomial density is connected with $\mu _{ji}$ through the logit function.

### Prior setting

**Hierarchical structure for the factor loadings $W$** We consider the following hierarchical priors for $W$: 


\begin{align*}&\begin{aligned} &\log\pi(W|\lambda)= C + \sum_{j,l}\log\lambda_{jl}-\sum_{j,l}\lambda_{jl}\left\vert w_{jl}\right\vert, \\ &\log\pi(\alpha|\Omega)= C + \frac{L}{2}\log\left\vert\Omega\right\vert-\frac{1}{2\nu_2}\sum_{l}^{T}\widetilde{\boldsymbol{\alpha}}_l^T\Omega\widetilde{\boldsymbol{\alpha}}_l,\\ &\log\pi(\Omega) = C + \frac{\Delta}{2}\log\left\vert\Omega\right\vert - \frac{\eta}{2}\text{tr}(\mathcal{E}\Omega) - \infty\boldsymbol{1}(\Omega \notin \mathcal{M}_{\mathcal{G}}), \end{aligned}\end{align*}

where $\widetilde{\boldsymbol{\alpha }}_l = \boldsymbol{\alpha }_l-\nu _1\boldsymbol{1}$, $\Delta =\eta (1+\epsilon )$ and $\mathcal{E}=(\boldsymbol{11^T}+\epsilon I)$. $\mathcal{M_G}$ is the set of positive definite and symmetric matrices compatible with the graph $\mathcal{G}$. Each element of $W$ is assigned with a Laplacian prior, where $\lambda _{jl}$ refers to the individual penalty parameter that helps to achieve the adaptive shrinkage on the factor loadings. We further impose hierarchical priors on $\lambda $ to incorporate the graph knowledge. To this end, we employ a multivariate Gaussian prior for $\boldsymbol{\alpha }_l = (\alpha _{1l},\dots , \alpha _{pl})^T$ for $1\leq l \leq L$, where $\alpha _{jl} = \log \lambda _{jl}$ with the precision matrix $\Omega $, who is associated with a novel prior known as the graph-constrained Wishart prior. It is worth noting that such hierarchical prior indicates that the correlations between the shrinkage parameters are governed by the underlying graph knowledge. Specifically, the presence of edges are expected to impact the correlations, leading to a similar shrinkage level for connected features. Hence, these connected features tend to be simultaneously selected or excluded for within a bicluster. The parameters $\nu _1$, $\nu _2$, $\eta $ and $\epsilon $ can be either user-specified or selected through a tuning procedure. In particular, $\nu _1$ controls the overall *a priori* shrinkage level, while $\nu _2$ affects the adaptivity to this pre-specified level of shrinkage. When $\nu _2\to 0$, all shrinkage parameters $\lambda _{jl}$ will concentrated around $e^{\nu _1}$. Both $\eta $ and $\epsilon $ are positive and control the adaptivity to the data as well as the level of correlations among the shrinkage parameter, respectively.

The benefits of utilizing such a novel constrained Wishart prior in terms of variable selection are 2-fold compared with other graph-guided priors. First, unlike the MRF priors [23], which suffer from phase transition phenomenon, this novel prior can explicitly control the correlations between the shrinkage parameter through the parameter $\epsilon $. It is because the mode of the prior, without the graph constraints, is $(\frac{\boldsymbol{1}\boldsymbol{1}^T+ \epsilon I}{1+\epsilon })^{-1}$, implying that the off-diagonal entries of $\Omega ^{-1}$ are concentrated around $\frac{1}{1+\epsilon }$. Moreover, the parameter $\eta $ allows us to control over the level of adaptivity to this prior. Increasing $\eta $ results in the posterior of $\Omega $ being more dependent on the prior and less impacted by the data. Second, our prior allows for a more flexible matrix space that needs to be compatible with the underlying graph $\mathcal{G}$, as we do not require the diagonal-dominant structure defined in [19], which hinders the efficient utilization of graph information when the size of the pathways in the graph becomes larger.

**Hierarchical structure for the latent factor $Z$** We also pursue sparsity in the latent factor matrix to identify biclusters. Similar to the factor loadings $W$, we place a Laplacian prior for each entry of $Z$, while the associated individual shrinkage parameter $\delta _{li}$ has the gamma distribution 


\begin{align*}&\begin{aligned} &\log\pi(Z|\delta) = C + \sum_{i,l}\log \delta_{li} - \sum_{i,l}\delta_{li}\left\vert z_{li}\right\vert,\\ &\log \pi(\delta_{li}) = C + (\nu_4-1)\log \delta_{li} - \nu_3\delta_{li}. \end{aligned}\end{align*}

The shape parameter $\nu _4$ determines the overall sparsity level of $Z$ with a larger value of $\nu _4$ yielding a sparser $Z$. The rate parameter $\nu _3$ controls the adaptivity. As $\nu _3$ decreases, the prior densities of $\delta _{li}$ tend to have heavier tails, indicating that $\delta _{li}$ becomes more adaptive to $Z$. Both $\nu _3$ and $\nu _4$ need to be pre-specified.

Finally, the prior on the location vector $\boldsymbol{m}$ is the multivariate normal distribution with the prior variance $\sigma _{m_j}^2$ which is given by 


\begin{align*}& \log \pi(\boldsymbol{m}) = C- \frac{1}{2\sigma_{m_j}^2}\boldsymbol{m}^T\boldsymbol{m}. \end{align*}

## COMPUTATION

In this section, we develop an efficient MCMC algorithm to explore the full posterior distribution 


(2) \begin{align*}& \begin{aligned} \pi(\boldsymbol{m},W,Z,\alpha,\Omega|X) \propto & \pi(X|\boldsymbol{m},W,Z) \pi(\boldsymbol{m}) \pi(Z|\delta) \pi(\delta)\\ \times &\pi(W|\alpha)\pi(\alpha|\Omega)\pi(\Omega). \end{aligned}\end{align*}

Two data augmentation techniques are used to improve the MCMC efficiency. First, the augmentation of the Pólya Gamma latent variable $\rho _{ji}$ [20] for the discrete data $x_{ji}$ facilitates an unified likelihood for the different data types 


\begin{align*}& \log\pi(\boldsymbol{x}_j,\boldsymbol{\rho}_j|\boldsymbol{\mu}_j) = -\frac{1}{2}\sum_{i}\rho_{ji}(\mu_{ji}-\psi_{ji})^2+\sum_{i}\kappa_{ji}\mu_{ji} + \log\pi(\boldsymbol{\rho}_j), \end{align*}

where $\boldsymbol{x}_j$, $\boldsymbol{\rho }_j$ and $\boldsymbol{\mu }_j$ refer to the $j$th row of $X$, $\rho $ and $\mu $ respectively. The values of $\psi _{ji}$, $\kappa _{ji}$, $b_{ji}$ and the prior density $\pi (\boldsymbol{\rho }_j)$ vary with the type of $\boldsymbol{x}_j$ and are given in Table 1. Suppose $\Psi $, $\mathbf{K}$ and $\rho $ are $p \times n$ matrices with $\boldsymbol{\psi }_j$ ($\widetilde{\boldsymbol{\psi }}_j$), $\boldsymbol{\kappa }_j$ ($\boldsymbol{\widetilde{\kappa }}_j$) and $\boldsymbol{\rho }_j$ ($\widetilde{\boldsymbol{\rho }}_j$) referring to their $j$th row (column). $\mathcal{G}(\cdot ,\cdot )$ ($\mathcal{PG}(\cdot ,\cdot )$) is the gamma (Pólya gamma) distribution. Second, we augment the Laplacian variable $w_{jl}$ and $z_{li}$ to a scale mixture of normals [24], introducing the augmented variables $\tau _{jl}^w$ and $\tau _{li}^z$, to facilitate the use the Gibbs sampler. We refer Section 2.1 in the Appendix for the full description of the data augmentation. Note that while most parameters can be sampled with the Gibbs sampler, the transformed shrinkage parameter $\alpha $ has to be sampled via the Metropolis-Hastings (MH) algorithm.

**Table 1:** Likekihood for different data types and formula components of Pólya Gamma classes. $x_{ji}$, $n_j$ and $r_j$ refer to the data, the number of trials and the number of failures. $\mathcal{G}$ and $\mathcal{PG}$ are the gamma and Pólya Gamma distribution

Data	$\psi _{ji}$	$\kappa _{ji}$	$b_{ji}$	$\pi (\boldsymbol{\rho }_j)$
Gaussian	$x_{ji}$	0	NA	$ \mathcal{G}(\frac{\zeta _j}{2},\frac{\zeta _j}{2})$
Binomial	$0$	$x_{ji}-b_{ji}/2$	$n_j$	$\mathcal{PG}(b_{ji},0)$
Neg binomial	$0$	$x_{ji}-b_{ji}/2$	$x_{ji}+r_j$	$\mathcal{PG}(b_{ji},0)$

### Conditional posterior distribution

**The conditional distribution for $\Omega $**  $\Omega $ is sampled column by column with the block-wise Gibbs sampler. We first represent $\Omega $, $\Sigma =\Omega ^{-1}$ and $A=\eta (\boldsymbol{11^T}+\epsilon I)+\frac{1}{\nu _2}\sum _{l}(\boldsymbol{\alpha }_l-\nu _1\boldsymbol{1})(\boldsymbol{\alpha }_l-\nu _1\boldsymbol{1})^T$, after implicit row and column permutations, in the block forms, respectively, 


\begin{align*} & \begin{bmatrix} \Omega_{-j,-j} & \boldsymbol \omega_{-j,j}\\ \boldsymbol \omega_{-j,j}^T &\omega_{jj}\end{bmatrix},\quad \begin{bmatrix}\Sigma_{-j,-j} & \boldsymbol \sigma_{-j,j}\\ \boldsymbol \sigma_{-j,j}^T &\sigma_{jj}\end{bmatrix}, \quad \begin{bmatrix}A_{-j,-j} & \boldsymbol{a}_{-j,j} \\\boldsymbol{a}_{-j,j}^T &a_{jj}\end{bmatrix}, \end{align*}

where $\omega _{jj}$, $\boldsymbol \omega _{-j,j}$ and $\Omega _{-j,-j}$ refer to the $j$th diagonal element, the off-diagonal vector of the $j$th column $\boldsymbol \omega _j$ and the submatrix of $\Omega $ after removing the $j$th row and column. The block components of $\Sigma $ and $A$ can be interpreted in the same way. Next, we represent $\Omega _{-j,-j}$ and $ \Sigma _{-j,-j}$ with $$\begin{bmatrix} \widetilde{\Omega }^{11}_{j} & \widetilde{\Omega }^{10}_{j} \\ \widetilde{\Omega }^{01}_{j} & \widetilde{\Omega }^{00}_{j}\end{bmatrix}$$ and $$\begin{bmatrix} \widetilde{\Sigma }^{11}_{j} & \widetilde{\Sigma }^{10}_{j} \\ \widetilde{\Sigma }^{01}_{j} & \widetilde{\Sigma }^{00}_{j} \end{bmatrix}$$. The superscript $11$ ($00$) implies that the matrix is associated with the subvector of non-zero (zero) entries in $\boldsymbol \omega _{-j,j}$, denoted as $\boldsymbol \omega _j^1$ ($\boldsymbol \omega _{j}^0$). At the same time, $\boldsymbol{\sigma }^1_j$ ($\boldsymbol{\sigma }^0_j$) and $\boldsymbol{a}^1_j$ ($\boldsymbol{a}^0_j$) $\boldsymbol \omega _j^1$ ($\boldsymbol \omega _{j}^0$) in $\Sigma $ and $A$ are vectors associated with $\boldsymbol \omega _j^1$ ($\boldsymbol \omega _{j}^0$). The sampling of $\boldsymbol \omega _j$ can be factorized into the following two parts: 


(3) \begin{align*}& \begin{aligned} \boldsymbol \omega_j^1 | \Omega_{-j,-j},\alpha &\sim{\mathcal{N}}(\overline{\mu}_j,\overline{\Omega}_j/a_{jj}), \\ \xi_j | \Omega_{-j,-j},\alpha &\sim{\mathcal{G}} \left(\frac{\eta(1+\epsilon)+L}{2}+1,\frac{1}{2}a_{jj} \right), \end{aligned}\end{align*}

where $\xi _j = \omega _{jj} - (\boldsymbol \omega _j^1)^T \overline{\Omega }_j^{-1} \boldsymbol \omega _j^1$ with $\overline{\Omega }_j = \widetilde{\Omega }^{11}_j - \widetilde{\Omega }^{10}_j (\widetilde{\Omega }^{00}_j)^{-1} \widetilde{\Omega }^{01}_j$ and $\overline{\mu }_j = -\overline{\Omega }_j \boldsymbol{a}^1_j/a_{jj}$. The notation ${\mathcal{N}}(a,b)$ refers to the normal distribution with mean $a$ and covariance matrix $b$. The sample of $\omega _{jj}$ is obtained from $\xi _{j}$. It is worth noting that sampling $\boldsymbol \omega _j^1$ can be inefficient due to the computation of the inverse matrix $(\widetilde{\Omega }^{00}_j)^{-1}$, which is mitigated by introducing $\Sigma $. To be specific, we can avoid such inefficient computation if we update $\Sigma $ with the latest sample of $\Omega $ at each iteration due to the relationship $\overline{\Omega }_j^{-1} = \widetilde{\Sigma }^{11}_j - \boldsymbol \sigma _{j}^1(\boldsymbol \sigma _{j}^1)^T/\sigma _{jj}$. The updating strategy of $\Sigma $ is given by 


(4) \begin{align*}& \begin{aligned} \sigma_{jj} &\leftarrow \xi_j^{-1}, \\ \Sigma_{-j,-j} &\leftarrow \Omega_{-j,-j}^{-1} + \sigma_{jj} \Omega_{-j,-j}^{-1} \boldsymbol \omega_{-j,j} \boldsymbol \omega_{-j,j}^T \Omega_{-j,-j}^{-1}, \\ \boldsymbol \sigma_{-j,j} &\leftarrow -\Sigma_{-j,-j} \boldsymbol \omega_{-j,j} / \omega_{jj}. \end{aligned}\end{align*}

**The conditional distribution for $\rho $** The conditional posterior of $\rho _{ji}$ is the Pólya-Gamma (or Gamma) distribution for the discrete (Gaussian) data which is given by 


(5) \begin{align*}& \rho_{ji}\begin{cases} \sim \mathcal{PG}(b_{ji},\mu_{ji}), \\ \equiv \mathcal{G} \left( \frac{\zeta_j+n}{2},\frac{\zeta_j+\sum_i (x_{ji}-\mu_{ji})^2}{2} \right). \end{cases}\end{align*}

**The conditional distributions for $W$ and $\tau ^w$** Suppose $\boldsymbol{m}_j=m_j\boldsymbol{1} \in \mathbb{R}^{n\times 1}$ with $m_j$ as the $j$th entry of $\boldsymbol{m}$. $P_j$ is a diagonal matrix with $\boldsymbol{\rho }_j$ as its diagonal entries. The conditional distributions for the $j$th row of $W$  $\boldsymbol{w}_j$ and $(\tau _{jl}^{w})^2$ are given by 


(6) \begin{align*}& \begin{aligned} &\boldsymbol{w}_j^T|\boldsymbol{x}_j,Z,\boldsymbol{m}_j,\tau_{j1}^2,\dots,\tau_{jL}^2 \sim{\mathcal{N}}(\boldsymbol{a}_j,B_j),\\ &\frac{1}{\tau_{jl}^2}|\lambda_{jl} \sim \mathcal{IN}(\mu^{\prime}_{jl},\lambda^{\prime}_{jl}), \text{ for} l=1,\dots,L, \end{aligned}\end{align*}

where $\boldsymbol{a}_j = (ZP_jZ^T+\boldsymbol{D}_{\tau ^w}^{-1})^{-1}ZP_j(\boldsymbol{\psi }_j-\boldsymbol{m}_j+P_j^{-1}\boldsymbol{\kappa }_j)$, $B_j = (ZP_jZ^T+\boldsymbol{D}_{\tau ^w}^{-1})^{-1}$ with the diagonal matrix $\boldsymbol{D}_{\tau ^w}$, whose diagonal entries are the $j$th row of $\tau $, $\mu _{jl}^{\prime}=\left \vert \frac{\lambda _{jl}}{w_{jl}}\right \vert $ and $\lambda _{jl}^{\prime}=\lambda _{jl}^2$. $\mathcal{IN}(\mu ,\lambda )$ refers to the inverse-Gaussian distribution, whose density is defined in [25].

**The conditional distribution for $\alpha $** There is no close form for the conditional posterior of $\alpha _{jl}$ and we use the MH algorithm with the target distribution being 


(7) \begin{align*}& \pi(\alpha_{jl}|\Omega, \tau_{jl},\boldsymbol{\alpha}_{-j,l}) \propto \lambda_{jl}^2 e^{-\frac{\lambda_{jl}^2\tau_{jl}^2}{2}}e^{-\frac{1}{2\nu_2}\widetilde{\boldsymbol{\alpha}}_l^T\Omega\widetilde{\boldsymbol{\alpha}}_l},\end{align*}

where $\boldsymbol{\alpha }_{-j,l}=(\alpha _{1l},\dots ,\alpha _{j-1,l},\alpha _{j+1,l},\alpha _{pl})$. In the $t$th iteration, we generate the candidate sample $\alpha _{jl}^{(cand)}$ from the proposal distribution ${\mathcal{N}}(\alpha _{jl}^{(t-1)},q)$, where $q$ is a pre-specified variance and the acceptance probability is $\min (1,\pi (\alpha _{jl}^{(cand)}|\Omega , \boldsymbol{\tau }_{l},\boldsymbol{\alpha }_{-j,l}^{(t-1)})/\pi (\alpha _{jl}^{(t-1)}|\Omega , \boldsymbol{\tau }_{l},\boldsymbol{\alpha }_{-j,l}^{(t-1)}))$, where $\boldsymbol{\alpha }_{-j,l}^{(t-1)} = (\alpha _{1l}^{(t)}, \dots , \alpha _{j-1,l}^{(t)}, \alpha _{j+1,l}^{(t-1)}, \dots , \alpha _{pl}^{(t-1)})$.

**The conditional distribution for $Z$, $\tau ^z$ and $\delta $** Suppose $\widetilde{P}_i$ is a diagonal matrix with $\boldsymbol{\widetilde \rho }_j$ as its diagonal entries. The conditional distributions for the $i$th column of $Z$  $\boldsymbol{\widetilde z}_i$, $(\tau _{li}^{z})^2$ and $\delta _{li}$ are given by 


(8) \begin{align*}& \begin{aligned} &\boldsymbol{\widetilde z}_i| \boldsymbol{\widetilde x}_i,W,\boldsymbol{m},\boldsymbol{\tau}_i^z,\widetilde{P}_i,\boldsymbol{\xi}_i\sim{\mathcal{N}}(\boldsymbol{c}_{i},D_i),\\ &\frac{1}{(\tau_{li}^z)^2}|\delta_{li}, z_{li} \sim \mathcal{IG}(\mu^\dagger_{li},\lambda^\dagger_{li}), \\ & \delta_{li}|z_{li}, \tau_{jl}^{z} \sim \mathcal{G}\bigg(\nu_4+\frac{3}{2},\nu_3+\frac{(\tau_{jl}^{z})^2}{2}+\frac{z_{li}^2}{2(\tau_{jl}^{z})^2}\bigg), \end{aligned}\end{align*}

where $\mu ^\dagger _{li}=\left \vert \frac{1}{z_{li}}\right \vert $, $\lambda ^\dagger _{li}= \delta _{li}$, $\boldsymbol{c}_i =D_iW^T\widetilde{P}_i(\boldsymbol{\widetilde{\psi }}_i - \boldsymbol{m} + \widetilde{P}_i^{-1} \boldsymbol{\widetilde{\kappa }}_i)$, $D_i=(W^T \widetilde{P}_iW + (D_\xi )_i (D_{\tau ^z})_i^{-1})^{-1}$ with the diagonal matrix $(D_{\tau ^z})_i$ and $ (D_{\delta })_i$, whose diagonal entries are the $i$th columns of $\tau ^z$ and $\delta $ and $u_{il} = \frac{(\tau _{li}^{z})^2}{2}+\frac{z_{li}^2}{2(\tau _{li}^{z})^2}$.

**The conditional distribution for $\boldsymbol{m}$** The conditional posterior of $\boldsymbol{m}$ is the multivariate normal with each entry being conditionally independent, whose conditional distribution is given by 


(9) \begin{align*}& m_j |X,W,Z,\rho \sim{\mathcal{N}}\big((v_m(\sum_i \kappa_{ij}-\rho_{ji}(\boldsymbol{w}_j^T\widetilde{z}_i-\psi_{ji})),v_m\big),\end{align*}

where $v_m=(\frac{1}{\sigma _{m}^2}+\sum _{i}\rho _{ji})^{-1}$ is the variance.

The overall time complexity of the MCMC algorithm is $O(\widetilde{p}^2(p+e)+npL^2)$, where $\widetilde{p}=\max p_h$ for $1\leq h\leq H$ and $e$ refers to the number of edges.

### Tuning

We use a variant of deviance information criteria (DIC) [26] to select the tuning parameters 


(10) \begin{align*}& \text{DIC}(L,\theta) = -2\sum l(X,\widehat{\mu}) + 4\times \sum \big(l(X,\widehat{\mu})-E[l(X,\mu)]\big),\end{align*}

where $\theta $ is a subset of potential tuning parameters $(\nu _1,\nu _2,\nu _3,\nu _4, \epsilon ,\eta )$, $l(\cdot ,\cdot )$ refers to the log-likelihood and the mean of log-likelihood $E[l(X,\mu )]$ is calculated over the MCMC samples with $E[l(X,\mu )]=\frac{1}{T}\sum _{t} l(X,\mu ^t)$. $\widehat{\mu }$ is computed by $\frac{1}{T}\sum _{t} \mu ^{t}$ with $\mu ^{t}= m^{t} + W^tZ^{t}$, where the superscript $t$ refers to the $t$th MCMC samples. We conduct grid search for ($\nu _1$,$\nu _3$,$\nu _4,L$) and select the model with the smallest DIC. In the selected model, the estimation of the $l$th bicluster is determined by whether the posterior credible intervals of $w_{jl}$ or $z_{li}$, with a pre-specified coverage probability, cover zero. Specifically, when the posterior credible interval of $w_{jl}$ or $z_{li}$ covers zero, it implies the $j$th feature or $i$th sample is selected by the $l$th bicluster. In our simulation and real data analysis, we use the $95$% posterior credible interval.

## SIMULATION

In this section, we compare the performance of BGB to existing biclustering methods in two simulation settings. These methods include (i) GBC [19]; (ii) SSLB [5]; (iii) ISA [9] and (iv) iSSVD [7]. Methods (i) and (ii) are Bayesian factor-model-based biclustering methods. iSSVD is a frequentist biclustering method based on the sparse singular value decomposition. The ISA does not assume a model for the data matrix and finds biclusters by selecting rows and columns using a certain threshold. Except for iSSVD, which is implemented in *Python*, all other methods are implemented in *R*. In our simulation studies, we fix $\nu _2 =0.5$ to allow for reasonable prior variance of shrinkage parameters in factor loadings; $\eta =15$ and $\epsilon =0.1$ to impose rational dependence on the graph and prior correlation of the shrinkage parameters. The grid-search area for the other tuning parameters is as follows: $\nu _1\in \{-2,-1,0,1,2\} $, $\nu _3\{1,0.1,0.01\}$, $\nu _4\in \{1,2,8,16\}$ and $L\in \{L-3,L-2,L-1,L,L+1,L+2,L+3\}$. The Gelman–Rubin statistic [27] is used to determine the MCMC convergence for BGB. All simulation results are based on the $100$ repeated samplings. Additional simulation studies can be found in Section 3.3 of Supplementary Materials.

We use the following metrics to measure the similarity between the estimated biclusters and true biclusters: (i) relevance (Rel) and recovery (Rec) [28]; (ii) clustering error (CE) [29] and (iii) consensus score (CS) [3] (for more details, see Section 3 of the Supplementary Material). All of the metrics are constructed using the Jaccard index and range between $0$ and $1$. Rel measures how well the estimated biclusters represent the true biclusters on average, while Rec instead evaluates how similar the true biclusters are to the estimated biclusters on average. The first step to calculate CS and CA is matching the estimated biclusters and true biclusters by the Hungarian method [30]. Then, CS reports the maximum overlapping proportion between two groups of biclusters and CA, which is defined as $1-$CE, can be viewed as a weighted version of the CS, scaled by the bicluster size when calculating the overlaping proportions. Therefore, larger biclusters may have a greater impact on CA than CS.

We conducted simulation studies with $n=90$ samples, $p=300$ features and $L=6$ true biclusters in the non-overlapping scenario (**Simulation 1**) and overlapping scenario (**Simulation 2**), which is overlapped on the feature-wise with the size $15$. In each setting, we further consider four graph structures and two different data types: the Gaussian and binomial data. $W$ is a $p\times L$ matrix whose non-zero entry indices in each column range from $1+50(l-1)$ to $50l$ in **Simulation 1**, or from $1+48(l-1)$ to $\min \{48(l-1)+63,300\}$ in **Simulation 2**, for $1 \leq l\leq 6$. $Z$ is an $L\times n$ matrix with the same sparsity pattern in both settings. The non-zero entry indices in each row range from $1+15(l-1)$ to $15l$ with $1 \leq l\leq 6$. The non-zero entries of $W$ and $Z$ are generated from ${\mathcal{N}}(2,0.1^2)$ and randomly assigned to be positive or negative with the probability $0.5$. For the Gaussian data, we generate $X$ through ${\mathcal{N}}(WZ,4)$. For the binomial data, we generate $X$ through $\text{Binomial}(n_j,\frac{1}{1+e^{-WZ}})$, where the number of trial $n_j$ is randomly sampled from $10$ to $30$.

We consider four working graph structures, denoted as $\mathcal{G}_1$ to $\mathcal{G}_4$, all based on the structure of $W$. These graphs differ in their informativeness, which is determined by the types of edges they contain. Edges connecting nodes belonging to the same pathway are referred to as within-pathway edges, while edges connecting nodes from different pathways are labeled as across-pathway edges. Within-pathway edges are expected to provide useful information, whereas across-pathway edges are considered noise. Specifically, $\mathcal{G}_1$ represents the null graph with no edges. $\mathcal{G}_2$ consists of $6$ underlying pathways, corresponding to the columns of $W$, respectively, and contain only within-pathway edges. Each pathway is defined by the non-zero features of a certain column of $W$, making $\mathcal{G}_2$ the true graph that is compatible with the structure of $W$. $\mathcal{G}_3$ is constructed by adding across-pathway edges to $\mathcal{G}_2$ with a probability of $0.1$. Lastly, $\mathcal{G}_4$ is formed by replacing the within-pathway edges in $\mathcal{G}_2$ with across-pathway edges with a probability of $0.5$. For a visual representation of these graphs, please refer to Section 3 of the Supplementary Material.

Table 2 and 3 show that the proposed method (BGB) consistently outperforms the competitors in both simulation settings, according to various metrics. The incorporation of biological graph knowledge has proven to be effective in enhancing the performance of BGB across all cases. Most notably, the performance of BGB under $\mathcal{G}_3$, although not as strong as under $\mathcal{G}_2$ and $\mathcal{G}_4$, still surpasses the other methods and is better than under $\mathcal{G}_1$. This primarily demonstrates the robustness of the proposed method to noisy edges. All of methods, regardless of scenarios, tend to overestimate the number of biclusters $\widehat{L}$; however, the overestimation of BGB is within a quiet narrow margin compared with SSLB and iSSVD. Additionally, existing methods designed for continuous data yield poor performance on binomial data.

**Table 2:** Performance of BGB, GBC, SSLB, iSSVD and ISA for Gaussian and binomial data over replications in Simulation 1 (non-overlapping). Four graph structures $\mathcal{G}_i$ for $i=1,2,3,4$ are considered. Rec/Rel/CS/CA refers to the relevance/recovery/census score/clustering accuracy. $\widehat{L}$ is the estimated number of biclusters. Standard errors are shown in parentheses

Method	Rec	Rel	CS	CA	$\widehat{L}$
	Gaussian data				
BGB($\mathcal{G}_1$)	0.83(2.87E-02)	0.75(7.79E-02)	0.75(7.81E-02)	0.82(2.92E-02)	6.65(6.71E-01)
BGB($\mathcal{G}_2$)	0.90(2.10E-02)	0.85(6.97E-02)	0.87(6.98E-02)	0.89(2.18E-02)	6.40(5.03E-01)
BGB($\mathcal{G}_3$)	0.89(2.04E-02)	0.85(6.28E-02)	0.87(6.30E-02)	0.89(2.11E-02)	6.15(4.89E-01)
BGB($\mathcal{G}_4$)	0.89(1.97E-02)	0.87(5.51E-02)	0.85(5.51E-02)	0.89(1.95E-02)	6.35(3.66E-01)
GBC($\mathcal{G}_1$)	0.82(4.85E-02)	0.82(3.33E-02)	0.80(3.85E-02)	0.82(3.65E-02)	6.10(7.47E-01)
GBC($\mathcal{G}_2$)	0.86(3.99E-02)	0.84(3.75E-02)	0.83(1.99E-02)	0.84(6.65E-02)	6.20(3.47E-01)
GBC($\mathcal{G}_3$)	0.85(1.76E-02)	0.82(2.89E-02)	0.83(2.76E-02)	0.82(2.97E-02)	6.15(5.47E-01)
GBC($\mathcal{G}_4$)	0.84(5.76E-02)	0.82(3.97E-02)	0.82(6.76E-02)	0.80(1.97E-02)	6.20(6.47E-01)
SSLB	0.68(3.16E-01)	0.72(7.23E-01)	0.46(9.85E-01)	0.54(5.85E-01)	7.30(7.33E-01)
iSSVD	0.58(2.85E-01)	0.68(4.26E-01)	0.63(2.99E-01)	0.40(4.85E-01)	7.40(9.33E-01)
ISA	0.006(3.03E-04)	0.003(0.00E-04)	0.00(0.00E+00)	0.00(0.00E+00)	35.30(2.30E+00)
	binomial data				
BGB($\mathcal{G}_1$)	0.49(8.04E-02)	0.45(7.19E-02)	0.51(7.28E-02)	0.53(1.45E-02)	6.60(6.40E-01)
BGB($\mathcal{G}_2$)	0.82(8.39E-02)	0.63(7.92E-02)	0.62(8.29E-02)	0.75(1.07E-02)	6.20(1.03E-01)
BGB($\mathcal{G}_3$)	0.80(3.49E-02)	0.63(6.07E-02)	0.74(7.48E-02)	0.75(3.79E-03)	6.30(8.89E-01)
BGB($\mathcal{G}_4$)	0.78(3.23E-02)	0.58(5.14E-02)	0.76(5.88E-02)	0.70(5.60E-03)	6.50(6.16E-01)
GBC($\mathcal{G}_1$)	0.52(5.85E-02)	0.45(2.33E-02)	0.45(2.85E-02)	0.51(3.65E-02)	7.10(5.47E-01)
GBC($\mathcal{G}_2$)	0.81(4.29E-02)	0.63(3.25E-02)	0.57(1.98E-02)	0.67(6.65E-02)	6.85(3.47E-01)
GBC($\mathcal{G}_3$)	0.82(1.76E-02)	0.62(2.80E-02)	0.57(7.76E-02)	0.68(2.97E-02)	6.45(5.40E-01)
GBC($\mathcal{G}_4$)	0.76(3.36E-02)	0.55(1.97E-02)	0.55(3.26E-02)	0.63(1.97E-02)	6.70(3.80E-01)
SSLB	0.23(5.16E-02)	0.14(4.23E-02)	0.21(4.85E-01)	0.10(2.85E-02)	14.20(2.33E+01)
iSSVD	0.28(4.85E-02)	0.22(2.26E-02)	0.25(4.99E-01)	0.10(1.85E-02)	15.10(1.35E+01)
ISA	0.01(9.23E-03)	0.00(0.00E+00)	0.00(0.00E+00)	0.00(0.00E+00)	55.30(2.70E+00)

**Table 3:** Performance of BGB, GBC, SSLB, iSSVD and ISA for Gaussian and binomial data over replications in Simulation 2 (overlapping). Four graph structures $\mathcal{G}_i$ for $i=1,2,3,4$ are considered. Rec/Rel/CS/CA refers to the relevance/recovery/census score/clustering accuracy. $\widehat{L}$ is the estimated number of biclusters. Standard errors are shown in parentheses

Method	Rec	Rel	CS	CA	$\widehat{L}$
	Gaussian data				
BGB($\mathcal{G}_1$)	0.84(3.02E-02)	0.81(6.05E-02)	0.81(6.06E-02)	0.84(3.18E-02)	6.25(4.44E-01)
BGB($\mathcal{G}_2$)	0.87(2.67E-02)	0.84(7.73E-02)	0.84(7.73E-02)	0.87(3.05E-02)	6.30(5.71E-01)
BGB($\mathcal{G}_3$)	0.88(2.57E-02)	0.84(6.36E-02)	0.84(6.37E-02)	0.88(2.64E-02)	6.30(5.71E-01)
BGB($\mathcal{G}_4$)	0.85(2.73E-02)	0.84(5.74E-02)	0.84(5.74E-02)	0.86(3.05E-02)	6.15(4.89E-01)
GBC($\mathcal{G}_1$)	0.80(4.11E-02)	0.80(5.23E-02)	0.79(3.11E-02)	0.81(4.22E-02)	6.10(2.50E-01)
GBC($\mathcal{G}_2$)	0.84(3.98E-02)	0.82(3.08E-02)	0.81(2.98E-02)	0.82(3.98E-02)	6.10(9.88E-02)
GBC($\mathcal{G}_3$)	0.84(7.12E-02)	0.81(3.72E-02)	0.81(5.12E-02)	0.82(3.66E-02)	6.15(3.01E-01)
GBC($\mathcal{G}_4$)	0.82(2.10E-02)	0.81(2.56E-02)	0.80(7.11E-02)	0.81(4.53E-02)	6.20(2.75E-01)
SSLB	0.59(4.06E-01)	0.40(5.09E-01)	0.43(6.77E-01)	0.45(5.03E-01)	7.20(9.76E-01)
iSSVD	0.55(5.13E-01)	0.50(4.16E-01)	0.40(2.58E-01)	0.39(4.65E-01)	7.40(8.26E-01)
ISA	0.003(4.14E-04)	0.002(1.01E-04)	0.00(0.00E+00)	0.00(0.00E+00)	40.10(4.00E+00)
	binomial data				
BGB($\mathcal{G}_1$)	0.50(6.87E-02)	0.50(7.10E-02)	0.59(7.21E-02)	0.43(1.27E-02)	6.60(4.47E-01)
BGB($\mathcal{G}_2$)	0.80(4.88E-02)	0.77(4.71E-02)	0.76(6.08E-02)	0.66(2.68E-03)	6.30(7.33E-01)
BGB($\mathcal{G}_3$)	0.77(4.07E-02)	0.76(5.65E-02)	0.76(6.10E-02)	0.66(9.86E-03)	6.30(4.47E-01)
BGB($\mathcal{G}_4$)	0.73(6.38E-02)	0.71(7.06E-02)	0.71(7.45E-02)	0.62(1.22E-02)	6.20(6.16E-01)
GBC($\mathcal{G}_1$)	0.54(3.11E-02)	0.56(7.23E-02)	0.57(4.11E-02)	0.45(5.22E-02)	6.60(5.50E-01)
GBC($\mathcal{G}_2$)	0.75(3.90E-02)	0.72(3.68E-02)	0.67(3.98E-02)	0.63(3.08E-02)	6.45(3.88E-01)
GBC($\mathcal{G}_3$)	0.73(5.12E-02)	0.73(3.12E-02)	0.65(6.12E-02)	0.63(3.76E-02)	6.15(3.01E-01)
GBC($\mathcal{G}_4$)	0.70(3.10E-02)	0.67(2.96E-02)	0.63(8.11E-02)	0.57(4.50E-02)	6.20(6.75E-01)
SSLB	0.19(1.06E-02)	0.15(1.09E-02)	0.10(2.77E-02)	0.07(1.03E-02)	17.30(9.76E-01)
iSSVD	0.15(2.13E-02)	0.12(1.16E-02)	0.10(1.58E-02)	0.05(1.65E-02)	16.40(8.26E-01)
ISA	0.01(4.01E-03)	0.01(5.61E-03)	0.00(0.00E+00)	0.00(0.00E+00)	58.30(3.10E+00)

## REAL DATA ANALYSIS

Alzheimer’s disease (AD) is an irreversible neurodegenerative disease caused by the deterioration of brain tissue, which results in accelerated cognitive decline relative to normal aging. While there exist treatments for enhancing impaired neuron systems, these treatments cannot effectively prevent the progression of cognitive decline. A major obstacle in developing an effective treatment for AD is the incomplete understanding of its pathological mechanism. Hence, it is of critical importance to investigate and comprehend the etiology of AD.

In this article, we conduct the real data analysis that integrated SNP and gene expression data from the Alzheimer’s Disease Neuroimaging Initiative (ADNI). The dataset comprises $n=435$ subjects from different phases of the ADNI study, including **ADNI GO**, **ADNI2** and **ADNI1**. Among the samples, there are 39 with AD, 126 with normal cognitive (CN) and 270 with mild cognitive impairment (MCI). This is served as the ground truth when computing the performance metrics. The gene expression data include continuous measurements for $20\,093$ unique genes. We first shrinkage our attention to the top $10\,000$ genes with the largest sample variance since AD is believed to be primarily related to a small number of genes. Next, we employ the high dimensional ordinary least-squares projection (HOLP) [31] to reduce the dimensionality from an ultra-high number of features to a more manageable scale. HOLP is a feature screening method possessing the sure screening property, which guarantees that the refined subset of features will preserve the active features of the true model with overwhelming probability. We applied HOLP on the reduced subset to select the top $10\%$ relevant genes, totaling $1000$ genes. The response variable used for HOLP is the Clinical Dementia Rating Scale Sum of Boxes (CDR-SB) score, a commonly used measurement to diagnose dementia of AD. The SNP data, viewed as binomial variables taking values from $\{0,1,2\}$, include the top $935$ SNPs related to AD based on existing genome-wide association studies (GWAS) of AD [32]. Similarly, we select the top $10\%$ SNPs based on their $P$-values. The grid-search area of BGB is as follows: $\nu _1\in \{-5,-2.5,-1,0,1,2.5,5\} $, $\nu _2\in \{0.5,2\} $, $\nu _3\{1,0.1,0.01,0.001\}$ and $\nu _4\in \{1,4,8,16,32\}$. The maximum number of biclusters is set as $6$ for all methods to ensure a fair comparison.

Table 4 reports CS and CE for different methods. Both BGB and GBC are evaluated with (denoted with the superscript *) or without graph knowledge. The results in Table 4 show that BGB with the graph incorporation compares favorably with existing methods in terms of CS and CA, demonstrating the benefits of using the graph knowledge. GBC, another method that is able to incorporate the graph information, ranks as the second-best method. Furthermore, we conducted pathway enrichment analysis through **ToppGene Suite**, a one-stop portal for the pathway enrichment analysis, to explore the biological potential of the proposed method. A pathway is said to be enriched in a set of genes if this set of genes is found to be over-represented in the larger set of genes constituting the pathway. With the false discovery rate threshold of $0.05$, we found four statistically significant enriched pathways with the genes selected by the proposed method: reactome sensory perception pathway, KEGG olfactory transduction, reactome ligand-receptor interactions pathway, and WP eicosanoid metabolism via cyclooxygenases cox pathway, whose $P$-values are $7.5 \times 10^{-5}$, $2.2 \times 10^{-4}$, $2.6\times 10^{-4}$ and $2.2\times 10^{-4}$, respectively. All of these enriched pathways have been demonstrated to be closely related to the AD [33–35].

**Table 4:** Performance of different methods on ADNI dataset. Rec/Rel/CS/CA refers to the relevance/recovery/census score/clustering accuracy. The superscript ‘*’ refers to ’with the incorporation of graph’

	BGB	BGB*	GBC	GBC*	SSLB	ISA	iSSVD
CA	0.251	0.272	0.247	0.261	0.166	0.110	0.144
CS	0.210	0.261	0.213	0.237	0.186	0.066	0.149

## DISCUSSION

In this article, we propose a novel Bayesian biclustering method, aiming at grouping features and samples simultaneously. On the one hand, the proposed method enables the integrative analysis of multi-omics data that may consist of different data types, which allows for a comprehensive understanding of complex biological systems. On the other hand, we develop a novel prior to exploit the underlying biological graph information, leading to improved clustering performance. Extensive simulation studies and real data analysis demonstrate the superior performance and robustness of our proposed method, even in the presence of noisy graphs. The application of BGB extends to disease subtyping studies and other multi-modality learning tasks.

There are several potential extensions for future work. First, it would be interesting to extend the model to accommodate model selection procedure in a fully Bayesian framework. This will help determine the optimal model complexity and improve the interpretability of the results. Second, it is worthwhile to explore algorithmic optimizations and leverage parallel computing techniques to enhance the computational efficiency of our method. Furthermore, expanding the scope of our model to address more complicated problems, such as the scenarios with complex graph structures, incomplete multi-omics data or biological data beyond -omics data including imaging data and fMRI functional connectivity data, is another avenue of interest. Finally, motivated by the existing graph-knowledge-guided deep learning models [36–38], integrating the proposed graph-incorporated technique to the deep learning structures would be helpful to enhance the interpretability of the deep learning models.

**Key Points**

- We propose a novel knowledge-guided Bayesian biclustering method that can handle multiple modalities of different data types such as continuous and count data.
- A key feature of our method is its ability to incorporate biological graph knowledge effectively and flexibly. This feature enables our method to yield biologically more interpretable biclustering results that can shed insights into the molecular underpinnings of disease subtypes.
- Extensive numerical experiments have demonstrated our method’s superiority over several existing methods and its robustness when graphs used are noisy.

## Supplementary Material

supplementary_bbad446

## Data Availability

The source code is publicly available at https://github.com/littlekii/BGB/tree/main.

## Author Biographies

**Qiyiwen Zhang** is a postdoctoral in the Department of Biostatistics, Epidemiology, and Informatics at University of Pennsylvania.

**Changgee Chang** is an assistant professor in the Department of Biostatistics and Health Data Science at Indiana University School of Medicine.

**Qi Long** is a Professor in the Department of Biostatistics, Epidemiology, and Informatics at University of Pennsylvania.
